# Intermittent Gastro-Enteralgia

**Published:** 1831-04-01

**Authors:** 


### Case of Intermittent Gastro-Enteralgia. By M. Laborderie, M.D.

The narrator of this case was a stanch disciple of Broussais ; but, like many other disciples of great masters, was rather puzzled by the facts that presented themselves at the bedside of sickness !

*Case*. A young medical gentleman, aged 24 years, of nervous temperament, and subject to dyspepsia, had the imprudence to neglect his health, while pursuing his professional studies. In the Autumn of 1829, however, he retired to his friends in the country, hoping that change of air and relaxation from mental exertions might relieve the miseries of a disordered stomach. In vain! for now he began to fancy a number of ailments, in addition to the list of realities. The pain which he felt in his stomach after food, was construed into inflammation of the organ, and relays of leeches to the epigastrium, with absolute starvation were employed to remedy the dreaded evil. Under this plan the mind became more disturbed — sleep fled — strength decreased—and his embonpoint vanished. A young medical friend was consulted, who saw nothing but acute gastritis that had supervened on chronic. More leeches were applied, and the nutrition still further decreased. For a few days after this the epigastric pains were ameliorated ; but the strength was almost annihilated, and the emaciation made rapid advances. With the loss of blood the irritability of the nervous system became greatly heightened — and now a feature was added—an intermitting fever, of the tertian type, accompanied by a most distressing distention of the epigastric region. These paroxysms became more and more intolerable ; and the sulphate of quinine was proposed, but the patient refused, and sent for M. Laborderie. This gentleman examined the case with great attention, and although a stanch Broussaian himself, he could not but perceive that the disease of his young medical friend was rather gastralgia than gastritis. The acetate of morphine was prescribed, with gradually increasing diet, and at last some good old wine, much diluted with water. It is hardly necessary to state that, in a short time, the gastralgia, as well as the tertian symptoms became mitigated, and not long afterwards entirely disappeared.
—Revue Medicale.

